# Distinct Mechanisms of Sodium–Calcium Exchanger NCX1.4 Regulation by Ca^2+^, ATP, and PIP_2_

**DOI:** 10.3390/cells15181698

**Published:** 2026-09-19

**Authors:** Jennyfer Pastor, Moshe Giladi, Benny Da’adoosh, Tali Strauss, Bernard Attali, Daniel Khananshvili

**Affiliations:** 1 Department of Physiology and Pharmacology, Gray Faculty of Medical and Health Sciences, Tel Aviv University, Tel Aviv 69978, Israel; jennyfer@tauex.tau.ac.il (J.P.); moshegiladi@gmail.com (M.G.); talistrauss@mail.tau.ac.il (T.S.); battali@tauex.tau.ac.il (B.A.); 2 Internal Medicine D, Tel-Aviv Sourasky University Medical Center, Tel Aviv 6423906, Israel; 3 Blavatnik Center for Drug Discovery, Tel Aviv University, Tel Aviv 6997801, Israel; daadoosh@tauex.tau.ac.il

**Keywords:** sodium–calcium exchanger, NCX, ionic and metabolic regulation, ATP, PIP_2_

## Abstract

Mammalian Na^+^/Ca^2+^ exchangers (NCXs) mediate inward (Ca^2+^-exit) or outward (Ca^2+^-entry) currents (*I*_NCX_), with the peak current (*I*_peak_) declining to the steady-state current (*I*_ss_). The Ca^2+^-, ATP-, and PIP_2_-dependent activation of cardiac (NCX1.1) inward *I*_NCX_ has been previously documented. To investigate possible interactions among regulatory modes, we examined the effects of ATP and PIP_2_ on outward *I*_NCX_ when the CBD1 and CBD2 domains of NCX1.4 (brain variant) are Ca^2+^-occupied and -unoccupied. At nominally zero cytosolic Ca^2+^, ATP and PIP_2_ significantly reduced the outward *I*_peak_ amplitude. While increasing cytosolic Ca^2+^ increased *I*_peak_ and *I*_ss_/*I*_peak_, consistent with CBD1- and CBD2-dependent regulation, no significant effects of ATP or PIP_2_ were detected at 0.1–10 μM cytosolic Ca^2+^. These observations indicate distinct responses under the Ca^2+^ conditions examined and warrant direct mechanistic investigation. Although we observed opposing effects of ATP and PIP_2_ on inward (in NCX1.1) and outward (in NCX1.4) currents using different patch-clamp configurations and protocols, the data collectively suggest that Ca^2+^, ATP, and PIP_2_ regulatory modes may operate differently on the forward and reverse modes in NCX1.1 and NCX1.4 variants. Given the important physiological implications across distinct cell types (e.g., cardiomyocytes, neurons, glia, and kidney), careful investigation is needed to resolve how distinct regulatory modes affect the forward and reverse modes of mammalian NCX variants.

## 1. Introduction

**Figure 1:**
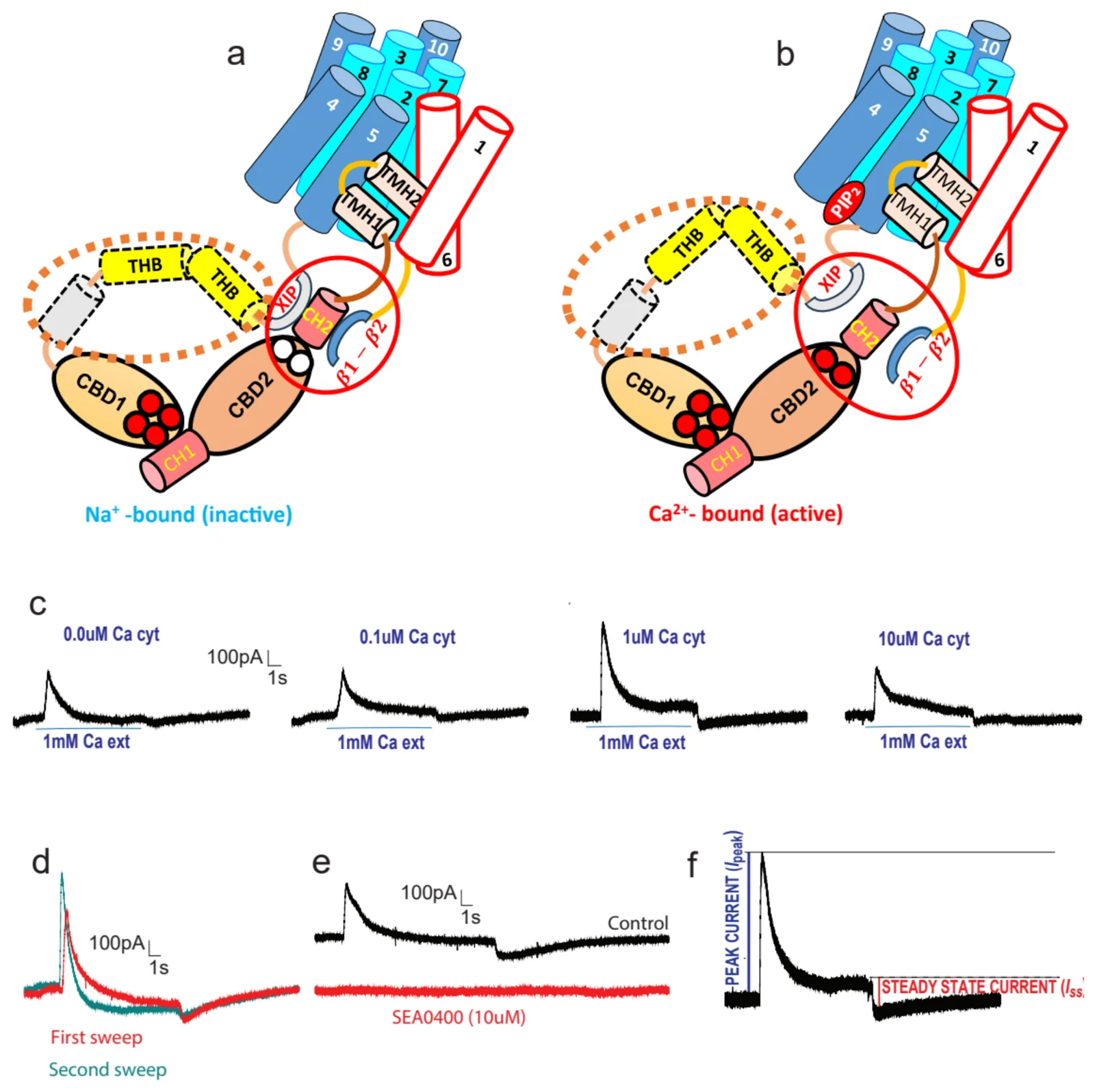
Overall structural and functional features of NCX1.4. A schematic illustration of the inactivation (**a**) and deactivation (**b**) assemblies, derived from cryo-EM structures of NCX1.1 [10] and NCX1.3 [11]. Structural elements not resolved by cryo-EM are shown with dashed lines. Cyan and white cylinders in panels A and B represent the ion-transporting helices (TM2, TM3, TM7, and TM8) and the two-helix sliding bundle (TM1/TM6), respectively. Red ellipses in A and B depict assembly and disassembly of the inhibitory complex (for more details, see the text). (**c**) Representative traces of NCX1.4 outward current triggered by 1 mM extracellular calcium for 10 s at the indicated fixed concentrations of cytosolic free Ca^2+^ (0, 0.1, 1.0, and 10 µM) in the whole-cell configuration of the patch-clamp technique (holding potential 0 mV). (**d**) Representative traces of NCX1.4-mediated outward currents obtained after multiple stimulations of the same cell with 1 µM cytosolic free Ca^2+^. The first (red) and second (green) sweep recordings indicate a change in the time-to-peak ratio. (**e**) The NCX1.4 outward current (black trace) is completely suppressed by 10 µM NCX1 inhibitor, SEA0400 (red trace). (**f**) Quantification of outward peak (*I*_peak_) and steady-state (*I*_ss_) currents. The peak current (in blue) is defined as the outward current measured relative to the baseline before initiating extracellular perfusion with 1 mM Ca^2+^. The steady-state (*I*_ss_) current (in red) is the outward current relative to the baseline after stopping the extracellular perfusion with 1 mM Ca^2+^. The steady-state current fraction is defined as *I*_ss_/*I*_peak_.

Tissue-specific isoforms of the Na^+^/Ca^2+^ exchanger (NCX1-3) and their splice variants regulate cellular signaling and help maintain ion homeostasis in excitable and non-excitable cells [1,2,3]. Like many other membrane proteins, NCX-mediated transport is allosterically regulated by ionic (Ca^2+^, Na^+^, and H^+^) and metabolic (ATP and PIP_2_) ligands [2,3,4,5,6,7]. The molecular mechanisms underlying the distinct regulatory modes and their interactions, which integrate NCX responses into various cellular signals, remain incompletely understood. This issue is of broad interest because NCX isoforms and splice variants (which are expressed in a tissue-specific manner) possess diverse regulatory features controlled by “small” (but specific) structural variances among NCXs [3,5,8,9]. Disease-related changes in tissue-specific expression and regulation of NCX variants contribute to cardiovascular and neurological disorders at cellular, organ, and systemic levels [2,3,5,7,8,9]. Selective pharmacological targeting of tissue-specific NCX variants could benefit many biomedical applications, although achieving this remains challenging. Eukaryotic and prokaryotic NCXs consist of ten transmembrane helices (TM1–TM10), and the transport sites for 3Na^+^ and 1Ca^2+^ are highly conserved [10,11,12,13]. Although NCX proteins share a common ion-transport mechanism [14,15,16,17,18,19], the ion-transport rates mediated by mammalian NCXs are 10^3^–10^4^ times faster than those of prokaryotic NCX_Mj for reasons that remain unclear [3,8,20,21,22,23,24,25]. In mammalian NCXs, the regulatory cytosolic 5L6 loop (between TM5 and TM6) contains an auto-inhibitory sequence (XIP) [26], a two-helix bundle (THB) [27], Ca^2+^ regulatory domains (CBD1 and CBD2) [28,29,30,31,32,33], and a PIP_2_-binding site [34] (Figure 1). Mammalian NCXs have three main modes of ion-dependent regulation [35,36,37,38,39]: (a) Ca^2+^-dependent increases in peak current, which occur upon Ca^2+^ binding to high-affinity sites of CBD1; (b) Na^+^-induced inhibition, caused by interactions of Na^+^ with an unidentified site; and (c) Ca^2+^-dependent relief (or alleviation) of Na^+^-induced inhibition, due to occupation of low-affinity Ca^2+^ sites on CBD2. The increase in peak current upon Ca^2+^ binding to CBD1 (K_0.5_ = 0.2–0.5 µM) is a common feature across NCXs [28,29,30,31,32,33]. Na^+^ induces a slow decline in the NCX peak current to steady-state levels [36,37,38,39,40]. Notably, Na^+^-induced inhibition is observed in NCX1 and NCX3, but not in NCX2 [28,29,30,31,32,33,37,38,39,40].

Biochemical tests [41,42,43,44,45,46,47,48] and structure-related biophysical studies [10,11,23,24,25,26,27,28,29,46] together with structure-based functional analyses [28,29,30,31,32,33,37,47,48] have shown that Ca^2+^ binding to CBD2 (K_0.5_ = 10–120 µM) alleviates Na^+^-induced inhibition of NCX1 and NCX3 expressed in oocytes [3,5,8,22]. However, careful examination of Ca^2+^ and ATP-dependent regulatory modes in BHK-expressed NCX1 and NCX3 [49] has shown that some regulatory modes that operate in mammalian cells (e.g., high-Ca^2+^-dependent slow inactivation of NCX) may be lost in excised patches and oocyte expression preparations [35,36,37,38,39,40]. Interestingly, exon-dependent structural variations determine the number of Ca^2+^ binding sites in CBD2, which ranges from zero to three and thus controls an alleviation of Na^+^-induced inhibition [3,8,28,29]. Notably, the kidney variant (NCX1.3) lacks Ca^2+^-dependent alleviation of Na^+^-induced inhibition because its exon composition precludes Ca^2+^ binding to CBD2 [29,40,44]. In contrast, the cardiac (NCX1.1) and brain (NCX1.4) variants exhibit Ca^2+^-dependent alleviation of Na^+^-induced inhibition, as in these splice variants of NCX1, exons place two Ca^2+^ sites in CBD2, which is absent in NCX1.3 [28,29,31,40,44].

The identity of the inhibitory Na^+^ site remains unknown [10,11,34,36], although Na^+^ does not compete with Ca^2+^ for interaction with CBD1 and CBD2 [31,45]. Notably, cryo-EM structures of NCX1.1 [10] and NCX1.3 [11] have been obtained in the presence of saturating Na^+^ and either residual Ca^2+^ (“inactivated”, Figure 1b) or high Ca^2+^ (“activated”, Figure 1b), with the high-affinity Ca^2+^ sites on CBD1 always occupied (Figure 1a,b). Therefore, detailed structural information on Ca^2+^-depleted (“fully inactivated”) NCX species is lacking. Cryo-EM structures indicate that the inactivation assembly (in Na^+^-bound species) forms a tightly packed four-stranded β-sheet (β-hub) that sandwiches the CH2 α-helix (Figure 1). The β1 and β2 (between TM1 and TM2) and β3 and β4 (including an auto-inhibitory XIP sequence at the C-terminus of TM5) sheets interact with the CH2 α-helix at the CBD2 tip (Figure 1a) [10,11]. Thus, Na^+^ may promote β-hub interaction with the cytosolic and membrane domains, thereby forming the inactivation assembly, whereas Ca^2+^-bound CBD2 destabilizes this structure (Figure 1a,b) [10,11,12].

Despite numerous attempts over the past 35 years, no physical evidence of NCX phosphorylation (by a protein kinase) was found. However, ATP-mediated phosphorylation of PI (by a lipid kinase) has been shown to activate inward NCX currents in excised and oocyte-expressed NCX1.1 preparations [4,6,10,34,47,48,49]. According to the cryo-EM structure of PIP_2_-bound NCX1.1, PIP_2_ interacts with the TM5 C-terminus at the cytosolic interface of TM4 and TM5 (near XIP) [34], suggesting that PIP_2_ may “stabilize” the CBD1-TM5 loop, leading to disassembly of the inactivation β-hub/CH2 complex.

The primary goal of this study was to examine the effects of ATP and PIP_2_ on NCX1.4 outward currents at different cytosolic Ca^2+^ concentrations. We therefore compared ATP- and PIP_2_-dependent effects under fixed concentrations of Ca^2+^, which probably produce differential occupancy of CBD1 and CBD2 by Ca^2+^, based on previously reported Ca^2+^ affinities to CBDs [28,29,30,31,32,33,34,35,36,37,38,39,40]. NCX1.4 was selected as an experimental model to examine mechanism-based interactions among distinct regulatory modes because these interactions may have physiological significance in glia-neuron interactions [7,8,9], which could be different from the cardiac NCX1.1, which has been the most extensively studied [2,3,4,5].

## 2. Materials and Methods

### 2.1. Drugs and Chemicals

Purified PI(4,5)P2 (Sino Biological, US Inc., Houston, TX, USA) was used in this study. Before the experiment, a 10 mM PIP_2_ stock solution (in DMSO) was diluted tenfold into buffer to 1 mM, briefly sonicated, and then diluted into patch-clamp solution to 10 µM. A 10 mM stock solution of the NCX inhibitor SEA0400 (Sigma-Aldrich, Rehovot, Israel) in DMSO was diluted into buffer to 0.5 mM with extensive vortex mixing, then diluted into patch-clamp solution to 10 µM. Nifedipine (Sigma-Aldrich, Rehovot, Israel) was then added to the extracellular solution to inhibit nonspecific Ca^2+^ currents; a 10 mM nifedipine (in DMSO) stock was added to the extracellular solution to give a final concentration of 10 µM. To inhibit Ca^2+^-dependent Cl^−^ channels, niflumic acid (Sigma-Aldrich, Rehovot, Israel) was also added to the extracellular solution to yield a final concentration of 100 µM.

### 2.2. Cell Culture

HEK293T cells were obtained from American Type Culture Collection (ATCC CRL-1573, Manassas, VA, USA) and maintained in DMEM (Sigma-Aldrich, Rehovot, Israel) supplemented with 10% fetal bovine serum (Sigma-Aldrich, Rehovot, Israel), 1% glutamine (Sigma-Aldrich, Rehovot, Israel), and 0.1% Pen-Strep solution (Sigma-Aldrich, Rehovot, Israel). For patch-clamp recordings, cells were detached with 0.25% trypsin for 30 s, centrifuged, and resuspended on a 13 mm #1 cover glass (Bar-Naor Ltd., Petach-Tikva, Israel) previously coated with 10 µg/mL poly-L-lysine for 20 min. Lipofectamine 3000 reagent (Thermo-Ficher, Raanana, Israel) was used for transient transfections of the plasmid vectors. HEK293T cells were used for patch-clamp recordings after 24 h of transfection.

### 2.3. DNA Cloning and Overexpression in HEK293T Cells

The DNA sequence of NCX1.4 (P23685) was introduced into the pcDNA3.4 expression vector, where a tobacco etch virus (TEV)-cleavable site was inserted between the given NCX sequence and the C-terminal 10xHis-tag. We codon-optimized the constructs to avoid negative cis-acting sites (such as splice sites and TATA boxes), prolong mRNA half-life, and maintain mRNA access to ribosomes, as previously reported [46]. Dr. Bernard Attali (Tel-Aviv University) provided the plasmid vectors bearing cDNA constructs encoding green fluorescent protein (GFP). The cloning of the NCX1.4 DNA construct has been previously described [36]. Cells were transfected 24 h before patch-clamp recordings with 0.5 µg of each cDNA per cover-glass and 0.3 µg of GFP cDNA. Typically, Lipofectamine 3000 achieved ~50% transfection efficiency.

### 2.4. Electrophysiology

We explored the whole-cell configuration for patch-clamp experiments by recording NCX1.4-mediated outward currents from HEK293T cells transfected for 24 h with the NCX1.4 DNA construct using Lipofectamine 3000 (see above). Data were sampled at 10 kHz and low-pass filtered at 4 kHz using a Multiclamp 700B amplifier, pClamp10 software, and a 4-pole Bessel low-pass filter (Molecular Devices, Sunnyvale, CA, USA). Patch electrodes were pulled from borosilicate glass pipettes (Warner Instruments, Hamden, CT, USA) with 3–5 MΩ resistance. Series resistances ranged from 8 to 12 MΩ and were compensated for in V-clamp recordings using the adjust-bottom Rs setting of the Multiclamp 700B amplifier (providing ≈85–90% compensation).

Patch pipettes were filled with the following internal solution: 120 mM NaCl, 2.5 mM KCl, 1 mM MgCl_2_, 12 mM TEA-Cl, 10 mM HEPES, 5 mM glucose, 100 µM niflumic acid, 5 mM EGTA (or 5 mM BAPTA, as indicated), and either 0.1 µM free calcium, 1 µM free calcium, or 10 µM free calcium, as calculated by MAXCHELATOR (WEBMAXC STANDARD) software 6.3, UCSF (https://somapp.ucdmc.ucdavis.edu/pharmacology/bers/maxchelator/webmaxc/webmaxcS.htm, accessed on 12 Setember 2026), and adjusted to pH 7.2 with tetramethylammonium hydroxide (Sigma-Aldrich, Rehovot, Israel). We adjusted the osmolality of the pipette solution to 300 mOsmol with sucrose and added 1 mM Na_2_ATP or 10 µM PIP_2_, as indicated in the figures. To calculate the desired free cytosolic Ca^2+^ concentrations, we adjusted the EGTA or BAPTA content in the pipette solution when ATP or PIP_2_ was present. The external solution contained 145 mM LiCl, 1 mM MgCl_2_, 10 mM glucose, and 10 mM HEPES, freshly supplemented with either 0.5 mM EGTA or 1 mM CaCl_2_, 10 µM Nifedipine (Sigma-Aldrich, Rehovot, Israel), and 100 µM NiCl_2_ (to inhibit nonspecific ion currents). We adjusted the pH to 7.4 with TMA and the osmolarity to 310 mOsmol with sucrose. Notably, the NiCl_2_ concentration used is at least 20–50 times lower than the concentration required for NCX inhibition. A concentration of 10 µM PIP_2_ was added to the pipette solution to assess the effect of cytosolic PIP_2_ on NCX1.4-mediated outward currents [34]. Notably, we avoided higher PIP_2_ concentrations because PIP_2_ micelles may form in the cytosol, affecting cell viability and whole-cell patch-clamp recordings. HEK293T cells transfected with NCX1.4 and GFP were held at 0 mV under voltage clamp. The NCX1.4 outward current was evoked by applying 1 mM Ca^2+^ to the extracellular solution for 10 s using a fast perfusion system.

To manage biological replicates, we used at least three batches of transfected cells in each experimental series. Only HEK293T cells with 15–35 passages were used for patch-clamp experiments, since cells with higher passage numbers exhibit altered background currents. We performed multiple patch-clamp measurements (*n* = 6–12) for each experiment for statistical analysis. We strictly controlled the timeline and stability parameters following membrane rupture (“break-in”) to achieve a stable equilibrium between the patch pipette and the cell interior. After break-in, we waited 2–5 min before executing our command protocols and performed at least two consecutive patch-clamp recordings to assess signal reproducibility. Matched vehicle concentrations of DMSO were added to control experiments to avoid uncertainties from nonspecific solvent effects.

### 2.5. Data Analysis

We analyzed data using Clampfit (pClamp10), Microsoft Excel (Microsoft, Richmond, WA, USA), and Prism 10.0 (GraphPad Software, Inc., San Diego, CA, USA). Results are expressed as means and standard errors of the mean (SEM). All treatments were conducted in independent cell groups. Statistical comparisons between two groups with Gaussian distributions were performed using a two-tailed unpaired t-test. For two unpaired groups with non-Gaussian distributions, comparisons were performed using the non-parametric Mann–Whitney test. For more than two groups, we used one-way ANOVA and the Kruskal–Wallis test, with post hoc tests. The statistical analyses reported here evaluate differences between experimental groups at individual cytosolic Ca^2+^ concentrations. No factorial interaction analysis between cytosolic Ca^2+^ concentration and ATP/PIP_2_ treatment was performed; therefore, differences in statistical significance between Ca^2+^ conditions were not interpreted as evidence of a statistical interaction.

## 3. Results

### 3.1. Patch-Clamp Recordings of HEK293T-Expressed NCX1.4

**Figure 2:**
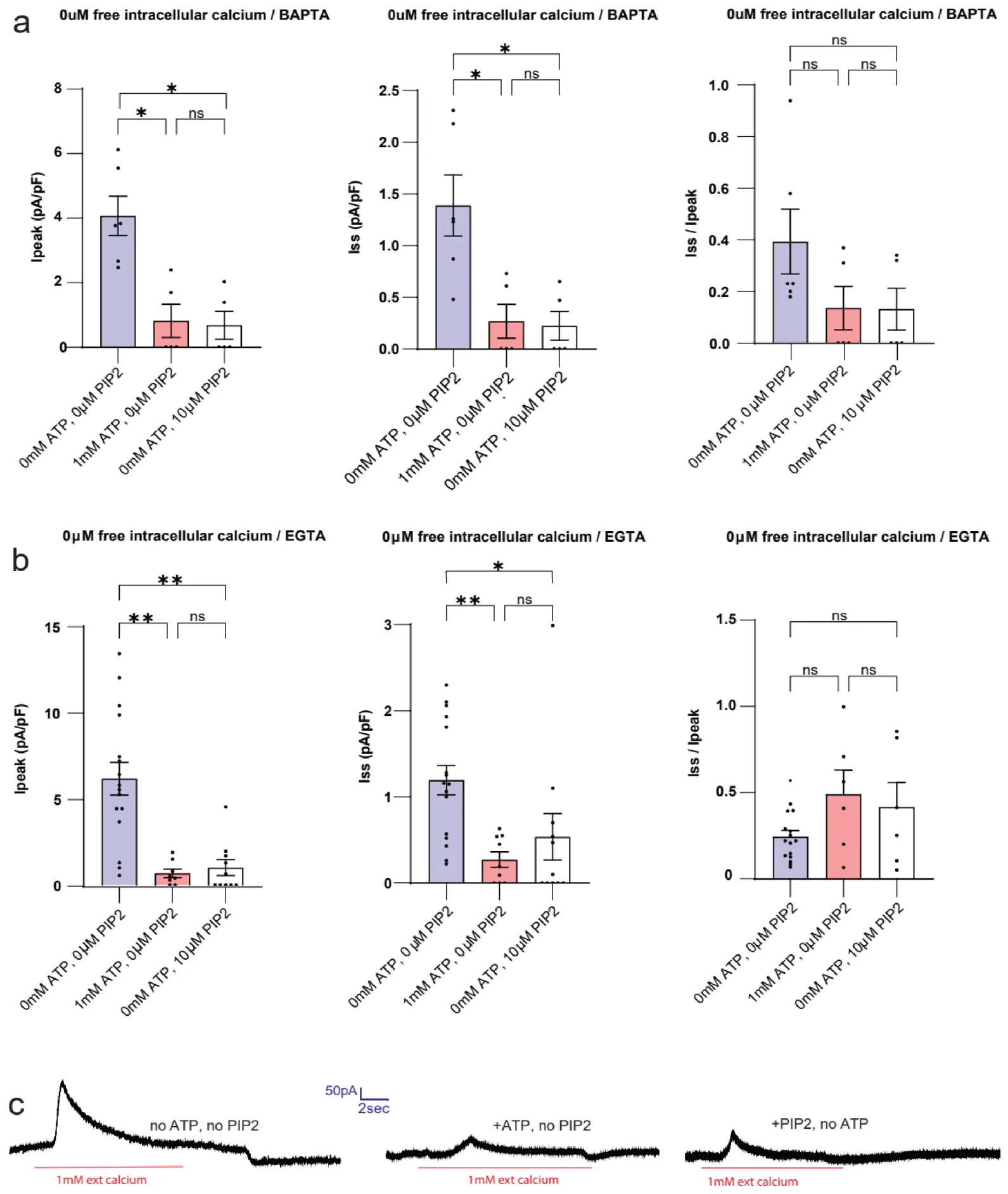
ATP or PIP_2_ reduces *I*_peak_ and *I*_ss_, but not *I*_ss_/*I*_peak_, when both CBDs are Ca^2+^-unoccupied at 0 µM cytosolic free Ca^2+^. NCX1.4 was activated with 1 mM extracellular Ca^2+^ for 10 s. (**a**) To ensure that free intracellular Ca^2+^ was tightly and rapidly controlled to near zero, we used 5 mM BAPTA instead of 5 mM EGTA. (**Left**) Without intracellular ATP or PIP_2_, NCX1.4 has an activity of 4.1 ± 0.6 pA/pF (*n* = 6). However, 1 mM ATP or 10 μM PIP_2_ significantly decreases the NCX1.4 activity to 0.82 ± 0.51 pA/pF (*n* = 5) and to 0.68 ± 0.43 pA/pF (*n* = 5), respectively; Kruskal–Wallis test, * *p* < 0.05. (**Middle**) Without intracellular ATP and PIP_2_, NCX1.4 has a steady-state current of 1.39 ± 0.29 pA/pF (*n* = 6), while in the presence of 1 mM intracellular ATP or PIP_2_, this decreases significantly to 0.27 ± 0.16 pA/pF (*n* = 5) and 0.22 ± 0.14 (*n* = 5), respectively; Kruskal–Wallis, * *p* < 0.05. (**Right**) Fractional activity of NCX1.4 steady-state currents without intracellular ATP and PIP_2_ (0.39 ± 0.12, *n* = 6), with 1 mM intracellular ATP (0.14 ± 0.83, *n* = 5) or with 10 μM PIP_2_ (0.13 ± 0.08, *n* = 5); Kruskal–Wallis test, *p* > 0.05. (**b**) NCX1.4 was activated by perfusion of 1 mM extracellular calcium for 10 s. Intracellular calcium was chelated with 5 mM EGTA. In the absence of ATP and PIP_2_, significant NCX1.4 activity was detected in the reverse mode with 6.2 ± 0.9 pA/pF and 1.2 ± 0.2 pA/pF peak (**left**) and steady-state currents (**middle**), respectively, producing a fractional activity (*I*_ss_/*I*_peak_) of 0.24 ± 0.03 (**right**) (*n* = 16). Adding 1 mM ATP to the patch pipette solution resulted in a significant decrease in the NCX1.4 peak (**left**) and steady-state (**middle**) outward currents (1.5 ± 0.9 pA/pF and 0.27 ± 0.09 pA/pF, *n* = 9, Kruskal–Wallis test: * *p* = 0.0109 and ** *p* = 0.0075). ATP did not significantly affect the fractional activity of the steady-state current (**right**). A similar effect was observed with 10 µM PIP_2_, with a significant decrease in the NCX1.4 peak (**left**) and steady-state (**middle**) outward currents (2.0 ± 1.1 pA/pF and 0.53 ± 0.26 pA/pF, respectively, *n* = 11; ** *p* = 0.0071 and * *p* = 0.0153). The fractional activity of the steady-state current was not altered by PIP_2_ (**right**). (**c**) Representative traces of NCX1.4-mediated outward currents with or without ATP or PIP_2_.

The reverse mode (Na^+^-dependent Ca^2+^ entry) of NCX1.4-mediated ion-exchange currents was measured by recording outward currents in a whole-cell patch-clamp configuration using HEK 293T cells expressing NCX1.4. In these experiments, the NCX outward current (the reverse mode of NCX) was recorded, representing an electrogenic stoichiometry (3Na^+^:1Ca^2+^) of ion exchange. These recordings account for Ca^2+^ influx into the cell when *E*_m_ > *E*_NCX_ [2,7,11] under conditions in which the cells are voltage-clamped at 0 mV. Outward currents of HEK293T-expressed NCX1.4 were recorded by rapidly switching the extracellular solution from 0.5 mM EGTA to 1 mM Ca^2+^ (Figure 1c). The NCX outward current, mediated by HEK293T-expressed NCX1.4, was validated by inhibiting *I*_NCX_ with 10 µM SEA0400 (Figure 1b), as previously shown for HEK293T-expressed NCX1.3 [11]. In this experimental setup, we voltage-clamped cells at a holding potential of 0 mV and elicited INCX outward currents by rapidly perfusing an extracellular solution containing 1 mM Ca^2+^ in the constant presence of 100 µM niflumic acid, a blocker of Ca^2+^-activated chloride channels. These channels are endogenously expressed in HEK 293T cells, and their presence can underestimate recorded outward currents by shifting the steady-state current baseline. ATP (1 mM) or PIP_2_ (10 µM) was added to the patch pipette intracellular solution, and free cytosolic Ca^2+^ concentrations were maintained at 0 µM (to keep CBD1 and CBD2 Ca^2+^ unoccupied), 1 µM (to allow Ca^2+^ binding to CBD1), or 10 µM (to occupy both CBDs, using 5 mM EGTA (Figure 1c,d) or 5 mM BAPTA (Figure 2).

The effects of cytosolic Ca^2+^ on peak current (*I*_peak_) and the steady-state current fraction (*I*_ss_/*I*_peak_) were investigated at four fixed Ca^2+^ concentrations (0, 0.1, 1.0, and 10 µM) in the absence of ATP or PIP_2_. Figure 1a shows that under these conditions, increasing the intracellular free Ca^2+^ concentration from 0 µM to 10 µM produces a bell-shaped effect on the peak and steady-state currents, with an initial increase up to 1 µM free Ca^2+^ followed by a reduced *I*_peak_ and an increased *I*_ss_/*I*_peak_ ratio at 10 µM free Ca^2+^. Similar bell-shaped Ca^2+^-dependent regulation has previously been observed in BHK-expressed NCX [49], but not in excised or oocyte-expressed preparations [32,33,34,35,36,37,38,39,40], suggesting that in mammalian cells, some additional regulatory modes may operate for NCX regulation [49].

Figure 1d shows that NCX1.4 outward currents could be elicited repeatedly in the same cell with reproducible magnitudes and that the time to peak was faster for the second external Ca^2+^ application than for the first. Figure 1e shows that SEA0400 inhibits the NCX1.4 outward currents, consistent with SEA0400’s known preference for inhibiting the reverse mode of NCX [37]. Notably, binding of SEA0400 to the TM domain of NCX1.1 and NCX1.3 stabilizes the exchanger in an inward-facing conformation, which may facilitate the inactivation assembly, which in turn may support Na^+^-induced inactivation of NCX1.1 and NCX1.3 [10,11,38]. Figure 1e shows how the peak current (*I*_peak_) and the fraction of steady-state current (*I*_ss_/*I*_peak_) were assigned and quantified. These parameters were chosen for current analyses of Ca^2+^ effects on HEK293T-expressed NCX1.4 (brain) (Figure 1c–f), because previous studies with the NCX1.1 (cardiac) and NCX1.3 (kidney) variants have shown that the increased *I*_peak_ and *I*_ss_/*I*_peak_ values represent the Ca^2+^ binding to CBD1 and CBD2, respectively [10,11,28,29,30,31,32,33,34,35,36,37,38,39,40].

### 3.2. Quantification of Outward I_peak_ and I_ss_ Currents of HEK293T-Expressed NCX1.4

It is noteworthy that the HEK293T-expressed NCX system fundamentally differs from the excised patch or oocyte-expressed NCX system, in which the fraction of slow Na^+^-induced inhibition (caused by Na^+^ addition) is defined as the ratio of the slowly declining inward peak current [10,32,33,34,35,36,37,38,39,40]. This definition of Na^+^-induced inhibition of *I*_NCX_ does not apply to the HEK293T-expressed NCX1.4 system because in our experimental system, the cytosolic Na^+^ concentration remains unchanged before and after extracellular perfusion with 1 mM Ca^2+^. Furthermore, some “nonspecific” currents may “shift” the baseline, thereby affecting the magnitude of the slow decline of *I*_NCX_ [49]. Thus, we evaluated the effects of Ca^2+,^ ATP, and PIP_2_ on *I*_peak_ and *I*_ss_ currents mediated by NCX1.4 expression in HEK293T cells, which represent the maximal and steady-state activities of NCX1.4-mediated ion exchange, respectively (Figure 1f). This is the most useful method of data analysis because, in our experimental system, the magnitude of the slow “inactivation current” (*I*_peak_ − *I*_ss_) and its fraction, (*I*_peak_ − *I*_ss_)/*I*_peak_, may include nonspecific outward currents.

In some recordings, the peak current of HEK293T-expressed NCX1.4 declined below the baseline when the holding voltage (e.g., +20 mV) exceeded the reversal potential, because Ca^2+^ entry (due to NCX activation) generates nonspecific ion currents (e.g., through Ca^2+^-activated Cl^−^ channels), thereby shifting the baseline, as suggested for BHK-expressed NCX1 and NCX3 [49]. We found that niflumic acid robustly and reproducibly reduced the baseline shift by inhibiting Ca^2+^-activated Cl^−^ channels. Regardless of the cause of the baseline shift, it is reasonable to define the peak current as the current relative to the baseline before NCX activation (*I*_peak_) and the steady-state current as the current relative to the baseline after NCX deactivation (*I*_ss_). To quantify the effects of distinct regulatory modes on slow *I*_NCX_ inactivation, we investigated how Ca^2+^, ATP, and/or PIP_2_ affect the fraction (activity) of the outward steady-state current (*I*_ss_/*I*_peak_).

### 3.3. ATP or PIP_2_ Significantly Reduces the NCX1.4-Mediated Currents at Nominally Zero Ca^2+^

First, we examined the effect of 0 µM free cytosolic Ca^2+^ (where both CBDs are unoccupied) on the regulatory modes of ATP and PIP_2_. Notably, in the absence of ATP and PIP_2_, significant NCX1.4 activity was observed in the reverse (outward current) mode with peak and steady-state currents of 6.2 ± 0.9 pA/pF and 1.2 ± 0.2 pA/pF, respectively, and a steady-state current fraction (*I*_ss_/*I*_peak_) of 0.24 ± 0.03 (Figure 2b,c; n = 16). A remarkable finding is that adding 1 mM ATP to the cytosolic solution significantly decreased the NCX1.4 outward peak amplitude, and steady-state currents were observed (Figure 2b,c; 1.5 ± 0.9 pA/pF and 0.27 ± 0.09 pA/pF, n = 9, Kruskal–Wallis test: * *p* = 0.0109 and ** *p* = 0.0075). However, ATP did not significantly affect the fractional activity of the steady-state currents (Figure 2b, right panel). A similar effect was observed with 10 µM PIP_2_, which significantly reduced the NCX1.4 peak and steady-state outward currents (Figure 2b,c; 2.0 ± 1.1 pA/pF and 0.53 ± 0.26 pA/pF, respectively, n = 11; ** *p* = 0.0071 and * *p* = 0.0153). Notably, PIP_2_ also did not alter the fraction of steady-state currents (Figure 2b, right panel). To ensure that free intracellular Ca^2+^ is tightly and rapidly controlled to near “zero” concentration, 5 mM BAPTA was used instead of 5 mM EGTA. Figure 2a shows that in the absence of ATP and PIP_2_, similar results are obtained with BAPTA at nominally 0 µM free intracellular Ca^2+^, yielding peak and steady-state currents of 4.0 ± 0.6 pA/pF and 1.3 ± 0.3 pA/pF, respectively (n = 6). Notably, both ATP and PIP_2_ significantly inhibit the *I*_peak_ of the NCX1.4 outward current (without altering the *I*_ss_/*I*_peak_ ratio) in the presence of 5 mM BAPTA (Figure 2a), consistent with the EGTA results.

### 3.4. ATP and PIP_2_ Do Not Affect the NCX1.4 Outward Currents 0.1–1 μM Ca^2+^

**Figure 3:**
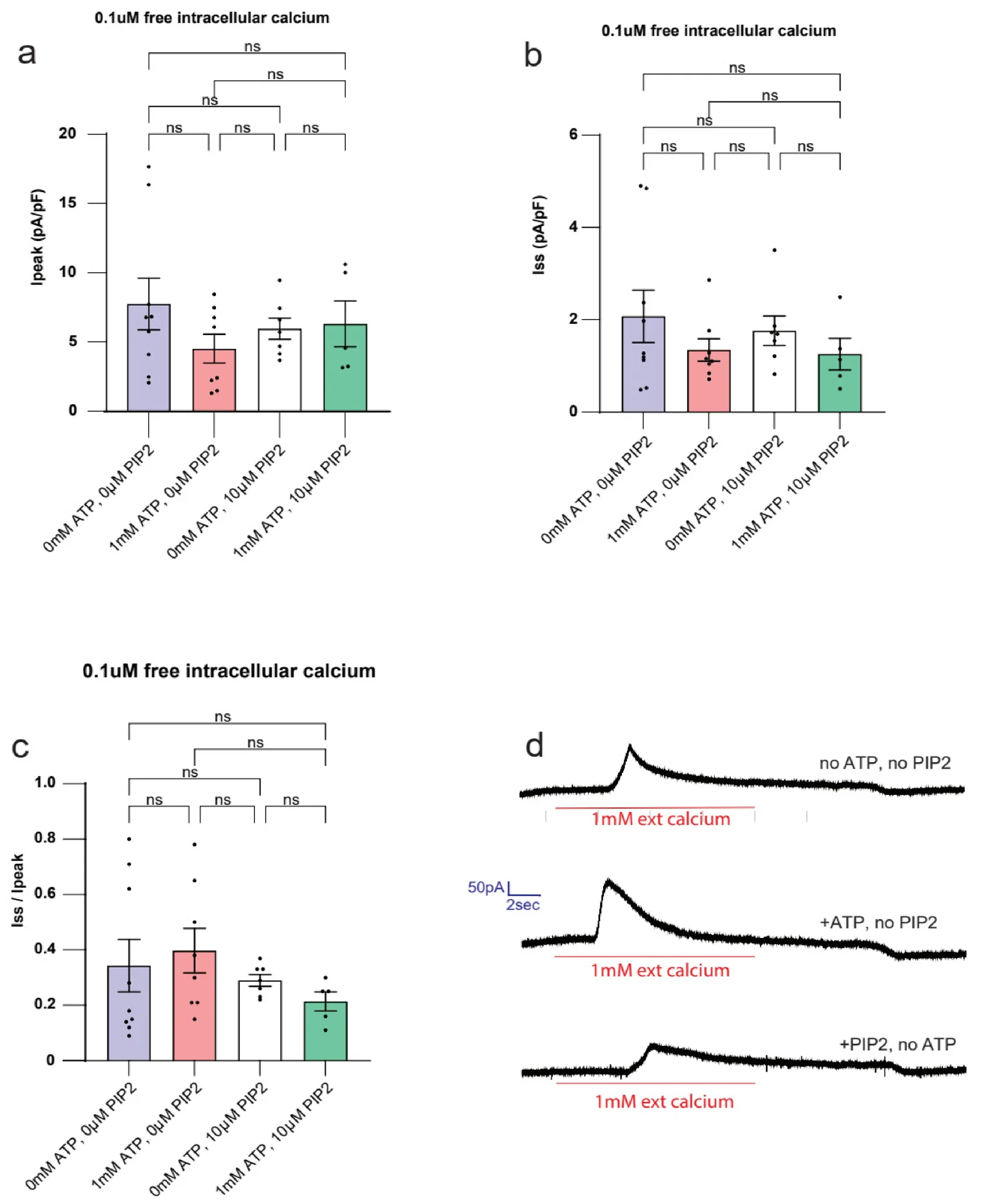
Effect of PIP_2_ or ATP on NCX1.4 outward currents at 0.1 μM cytosolic free Ca^2+^. NCX1.4 outward currents were elicited by rapid perfusion with 1 mM extracellular Ca^2+^ for 10 s. In contrast to conditions at 0 µM cytosolic Ca^2+^, at 0.1 µM cytosolic Ca^2+^, neither 1 mM ATP, 10 µM PIP_2_, nor 1 mM ATP + 10 µM PIP_2_ suppressed the outward NCX1.4 currents. At 0.1 µM cytosolic Ca^2+^, peak currents of 7.7 ± 1.8 pA/pF (**a**) and steady-state currents of 2.0 ± 0.5 pA/pF (**b**) were observed in the absence of ATP or PIP_2_ (*n* = 9). Neither ATP, PIP_2_, nor ATP+PIP_2_ had a significant effect on the *I*_peak_ (**a**), *I*_ss_ (**b**) or *I*_ss_/*I*_peak_ (**c**) parameters of NCX1.4-mediated outward currents. (**d**) Representative traces of NCX1.4 outward currents in the absence or presence of ATP or PIP_2_.

In contrast to the conditions at 0 µM free Ca^2+^ (when both CBDs are Ca^2+^-unoccupied), in the presence of 0.1 µM free intracellular Ca^2+^ (at which Ca^2+^ binds neither to CBD1 nor to CBD2), neither 1 mM ATP, 10 µM PIP_2_, nor 1 mM ATP+10 µM PIP_2_ decreased NCX1.4 outward currents (Figure 3a,b). Instead, they maintained the peak and steady-state currents at levels comparable to those observed at 0 µM free Ca^2+^ and with a similar fraction (*I*_ss_/*I*_peak_) of steady-state currents (7.7 ± 1.8 pA/pF and 2.0 ± 0.5 pA/pF for peak and steady-state currents (Figure 3a–c), respectively, in the absence of ATP or/and PIP_2_, n = 9; Figure 3a,b). These results suggest that a yet-unidentified high-affinity site (most likely not located on CBD1 or CBD2) may “abort” the inhibitory effects of ATP and PIP_2_ on NCX1.4-mediated outward currents at 0 µM free Ca^2+^ (Figure 2). Although Ca^2+^ has been reported to activate BHK-expressed NCX1 and NCX3 [49], the location and structural features of this activation remain unresolved. The putative site (most likely outside the CBDs) may “neutralize” the inhibitory effects of ATP or PIP_2_ that occur at zero Ca^2+^, although more dedicated, systematic studies are needed to address this peculiar issue.

**Figure 4:**
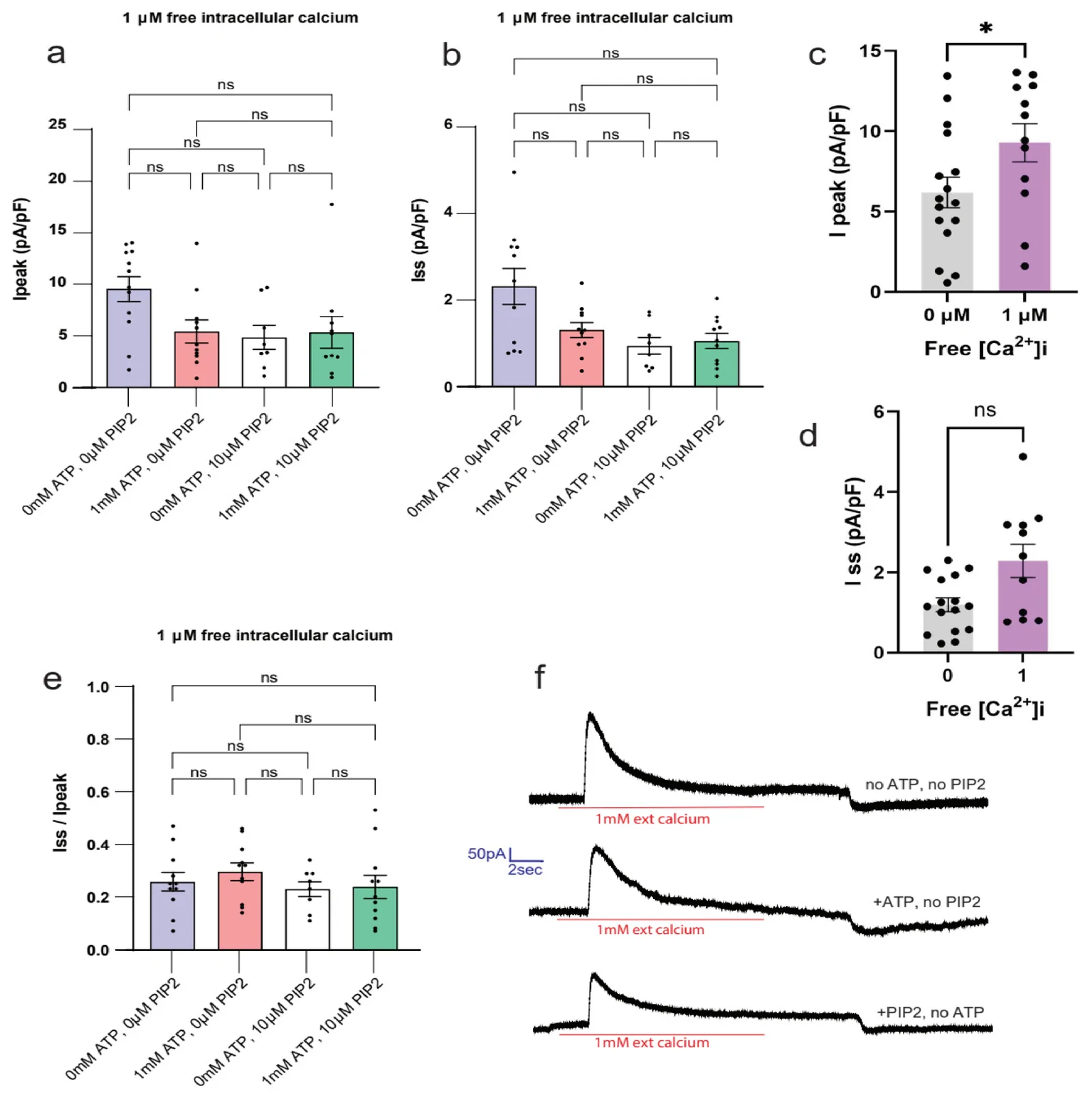
ATP and PIP_2_ do not affect NCX1.4 outward currents when Ca^2+^ binds to CBD1. NCX-mediated outward currents were measured at 1 µM free cytosolic Ca^2+^ in the presence or absence of 1 mM cytosolic ATP or 10 µM PIP_2_. (**a**,**c**): At 1 µM cytosolic Ca^2+^ and in the absence of ATP and PIP_2_, peak currents were significantly larger than those at 0 µM cytosolic Ca^2+^ (9.3 ± 1.2 pA/pF [*n* = 11] versus 6.2 ± 0.9 pA/pF [*n* = 16]; Mann–Whitney test * *p* = 0.0460). (**b**,**d**): Although steady-state currents at 1 µM Ca^2+^ were slightly larger than those at 0 µM Ca^2+^, these differences were not significant (2.28 ± 0.41 pA/pF [*n* = 11] versus 1.19 ± 0.17 pA/pF [*n* = 16]; Mann–Whitney test *p* = 0.0515). At 1 µM Ca^2+^, neither ATP, PIP_2_, nor ATP+PIP_2_ affected the peak (**a**) and steady-state currents (**b**) or the fraction (**e**) of steady-state currents (*I*_ss_/*I*_peak_) of the NCX1.4-mediated reverse (Ca^2+^-entry) mode. (**f**): Representative traces of NCX1.4-mediated currents in the absence or presence of ATP or PIP_2_.

Follow-up experiments were designed to determine how a probable occupancy of high-affinity Ca^2+^ sites on CBD1 influences ATP-dependent regulation of NCX1.4 at 1 µM cytosolic Ca^2+^. In these experiments, NCX-mediated outward currents were measured at 1 µM free cytosolic Ca^2+^ in the presence or absence of 1 mM cytosolic ATP and/or 10 µM PIP_2_. At 1 µM cytosolic Ca^2+^, in the absence of ATP and PIP_2_, peak currents were significantly larger than those at 0 µM cytosolic Ca^2+^ (Figure 4c,f; 9.3 ± 1.2 pA/pF [n = 11] versus 6.2 ± 0.9 pA/pF [n = 16]; Mann–Whitney test * *p* = 0.0460). The steady-state currents were also larger than those at 0 µM cytosolic Ca^2+^ but did not reach significance (Figure 4d,f; 2.28 ± 0.41 pA/pF [n = 11] versus 1.19 ± 0.17 pA/pF [n = 16]; Mann–Whitney test *p* = 0.0515). Under these conditions, neither ATP, PIP_2_, nor ATP+ PIP_2_ affected the peak or steady-state currents, nor did they affect the *I*_ss_/*I*_peak_ ratio of NCX1.4-mediated outward currents (Figure 4a–f).

### 3.5. ATP and PIP_2_ Do Not Affect the NCX1.4 Outward Currents at 10 μM Cytosolic Ca^2+^

**Figure 5:**
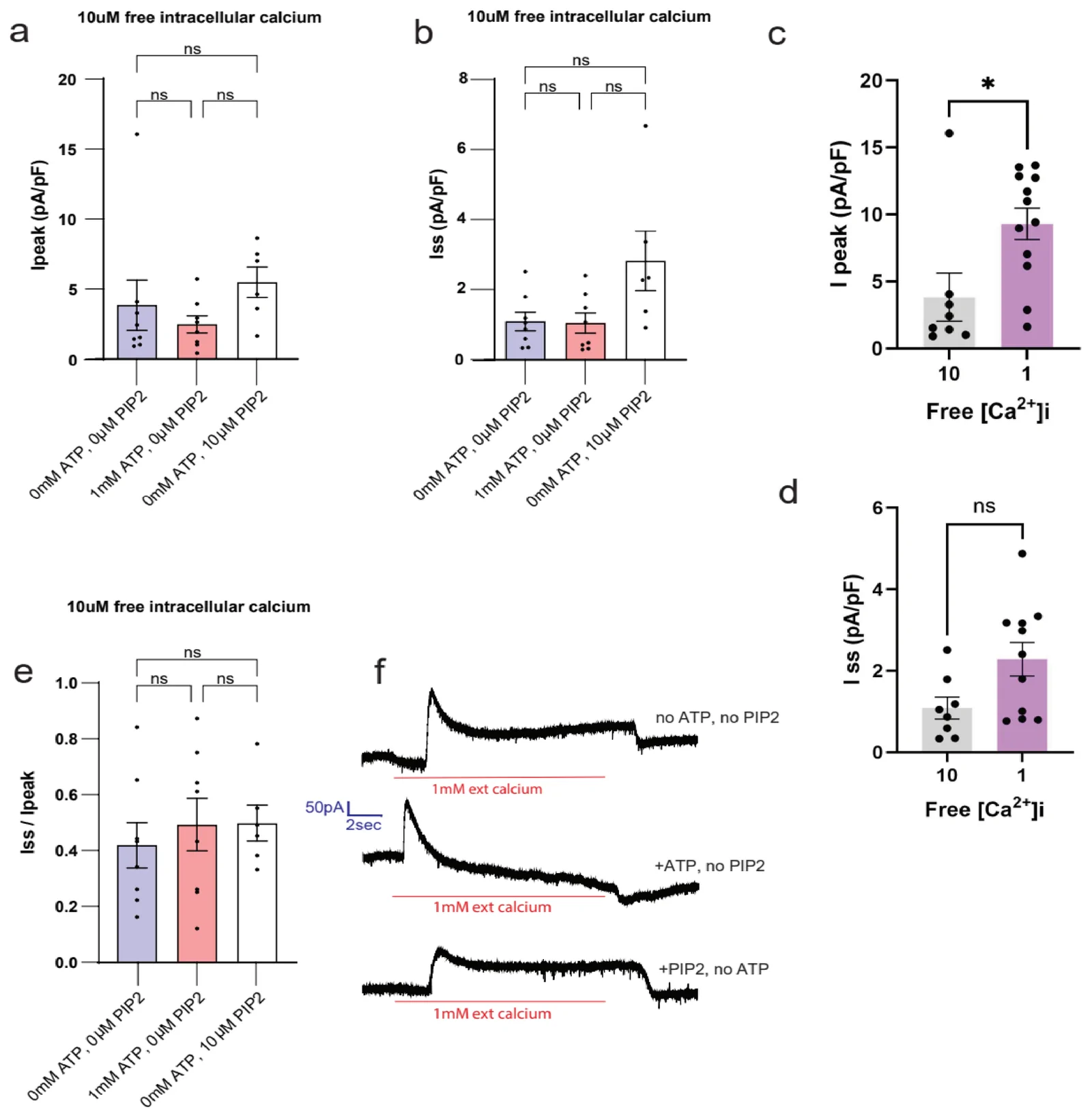
ATP and PIP_2_ do not affect NCX1.4 outward currents when Ca^2+^ occupies both CBDs. NCX1.4-mediated outward currents were measured at 10 µM free cytosolic Ca^2+^, under conditions in which Ca^2+^ occupies CBD1 and CBD2. (**a**,**c**) At 10 µM cytosolic Ca^2+^ and in the absence of ATP and PIP_2_, peak currents were significantly smaller than those at 1 µM cytosolic Ca^2+^ (3.8 ± 1.8 pA/pF [*n* = 8] versus 9.3 ± 1.2 pA/pF [*n* = 12]; Mann–Whitney test * *p* = 0.0159). (**b**,**d**) Although the steady-state currents recorded at 10 µM Ca^2+^ were somewhat lower than those at 1 µM cytosolic Ca^2+^, this difference was not significant (1.08 ± 0.26 pA/pF [*n* = 8] versus 2.28 ± 0.41 pA/pF [*n* = 11]; Mann–Whitney test *p* = 0.0620). At 10 µM cytosolic Ca^2+^, neither ATP nor PIP_2_ had a significant effect on the peak (**a**) or steady-state (**b**) currents. (**e**) At 10 µM Ca^2+^, the fraction of the steady-state current (*I*_ss_/*I*_peak_) was significantly larger than that observed at 1 µM Ca^2+^. (**f**) Representative traces of NCX1.4 outward currents in the absence or presence of ATP or PIP_2_.

To elucidate possible interactions among regulatory modes, we examined how ATP and PIP_2_ affect NCX1.4-mediated outward currents at 10 µM cytosolic Ca^2+^ under conditions in which both CBDs are Ca^2+^-bound (Figure 5). At 10 µM cytosolic Ca^2+^, in the absence of ATP and PIP_2_, peak currents were significantly smaller than those at 1 µM cytosolic Ca^2+^ (Figure 1c and Figure 5c; 3.8 ± 1.8 pA/pF [n = 8] versus 9.3 ± 1.2 pA/pF [n = 12]; Mann–Whitney test * *p* = 0.0159). These decreased peak currents cannot be explained by “nonspecific” ion currents contributing to NCX1.4 outward current recordings because, in the presence of the Ca^2+^-dependent Cl^−^-channel blocker, we obtained reasonably good baselines after stopping extracellular Ca^2+^ perfusion (Figure 5d). Three major factors may contribute to the observed decrease in *I*_peak_ at 10 µM vs. 1 µM: (a) reduced driving force for Ca^2+^ entry due to increased cytosolic Ca^2+^; (b) more cytosolic Ca^2+^ competes with Na^+^ for transport sites (with K_d_ in the range of 10–20 µM), thereby decreasing the driving force for Ca^2+^ entry; and (c) more cytosolic Ca^2+^ may occupy a putative inhibitory allosteric site (the structural identity of which has not yet been documented), thus decreasing *I*_peak_.

Notably, in the absence of ATP and PIP_2_, the fraction of steady-state currents (*I*_ss_/*I*_peak_) was considerably higher at 10 µM cytosolic Ca^2+^ (Figure 5e) than at 1 µM cytosolic Ca^2+^ (Figure 4e). Importantly, this change was accompanied primarily by a decrease in *I*_peak_ rather than a significant increase in *I*_ss_. Thus, the higher *I*_ss_/*I*_peak_ ratio is compatible with previously described CBD2-dependent modulation of NCX inactivation [10,11,34]. However, it is unclear whether increased *I*_ss_/*I*_peak_ reflects Ca^2+^-dependent alleviation of Na^+^-induced inhibition (due to Ca^2+^ binding to CBD2) [5,6,10,28,29,30,31,32,33,34,35,36,37,38,39,40] or whether an alternative mechanism (e.g., Ca^2+^-induced slow inactivation of NCX caused by Ca^2+^ interaction with a yet unknown site) may operate in a mammalian cell environment [49].

At 10 µM cytosolic Ca^2+^ (when both CBDs are occupied by Ca^2+^), neither ATP nor PIP_2_ affects the NCX1.4-mediated outward peak current (Figure 5a), steady-state current (Figure 5b), or the fraction (*I*_ss_/*I*_peak_) of steady-state current (Figure 5e). These findings are again consistent with the common mechanism of Ca^2+^-dependent regulation of mammalian NCXs [3,5,8,31,32,33], in which Ca^2+^ binding to CBD1 increases *I*_peak_, while subsequent Ca^2+^ binding to CBD2 increases *I*_ss_/*I*_peak_. Notably, at 0.1–10 µM free cytosolic Ca^2+^, we detect no significant effect of cytosolic ATP or PIP_2_ addition on NCX1.4-mediated outward currents (Figure 3, Figure 4 and Figure 5). Thus, the observed ATP- or PIP_2_-dependent inhibition of the NCX1.4 outward *I*_peak_ at zero Ca^2+^ (Figure 2) and the lack of ATP or PIP_2_ effect on NCX1.4-mediated outward currents at 0.1–10 µM Ca^2+^ (Figure 3, Figure 4 and Figure 5) fundamentally differ from the activating effects of ATP or PIP_2_ observed for NCX1.1-mediated inward currents, where activation of NCX1.1 was observed at 0.5–12 µM Ca^2+^ [10,34,47,48,49]. Notably, the electrochemical gradient for NCX-mediated ion exchange is much higher at <0.1 µM Ca^2+^ than at >0.5 µM Ca^2+^, which explains (at least partially) why relatively high values of *I*_peak_ were observed at zero Ca^2+^ in our experiments (Figure 2). Thus, it remains unclear whether previous NCX1.1 experiments adequately tested “zero” Ca^2+^ conditions used for NCX1.4 analysis. Additional experiments may be required to resolve these uncertainties.

## 4. Discussion

The recently determined cryo-EM structures of NCX1.1 [10,34] and NCX1.3 [11] offer new opportunities to understand how cytosolic ions (Na^+^, H^+^, and Ca^2+^) and metabolic ligands (ATP and PIP_2_) regulate NCX. Previous studies on BHK cells expressing NCX1 and NCX3 have shown that the ionic and metabolic modes and their interactions are more complex in mammalian cells than previously thought, because some regulatory modes may be absent in oocyte-expressed and excised-patch NCX preparations [49]. Interestingly, recent structure–function analyses of single-point mutations in NCX1.3 (using the HEK293T expression system) have shown that interactions between XIP and neighboring domains contribute to slow inactivation of HEK293T-expressed NCX1.3 [11]. However, it remains unclear how Na^+^ interacts with NCX to cause inactivation and how Ca^2+^ can alleviate Na^+^-induced inhibition. The situation becomes even more complicated when considering how ion-dependent and ATP/PIP_2_ regulatory modes integrate into cell-specific transitions and signaling [3,4,5,8,9,10,11]. In the present study, we tested how ATP and PIP_2_ affect the peak (*I*_peak_) and steady-state (*I*_ss_) outward currents of the HEK293T-expressed NCX1.4 (brain) variant at fixed cytosolic Ca^2+^ (0, 0.1, 1.0, and 10 µM), aiming to assess their effects under conditions in which both CBDs are Ca^2+^-occupied or unoccupied. Notably, a limitation of the present study is that we did not directly measure Ca^2+^ occupancy of CBD1 and CBD2; instead, we assumed probable occupancy states of CBD domains at the tested Ca^2+^ concentrations, based on reported Ca^2+^ affinities for CBD1 and CBD2 [28,29,30,31,32,33,34].

### 4.1. ATP and/or PIP_2_ Suppress the Outward Peak Current of NCX1.4 at “Zero” Cytosolic Ca^2+^

Notably, large outward *I*_NCX_ signals are observed at nominally “zero” cytosolic Ca^2+^ in the absence of cytosolic ATP or PIP_2_, with typical *I*_peak_ and *I*_ss_ values, suggesting that the NCX1.4-mediated Ca^2+^ entry mode can operate without any “activating” regulatory ligands (Figure 2). This observation can be explained by the very large electrochemical gradient (due to negligible cytosolic Ca^2+^ concentrations), which substantially accelerates NCX-mediated Ca^2+^ entry rates under the given experimental conditions. However, one striking finding is that at “zero” cytosolic Ca^2+^, when both CBDs are likely Ca^2+^-unbound, both ATP and PIP_2_ decrease the *I*_peak_ amplitude of outward *I*_NCX_, without significantly affecting the fraction (*I*_ss_/*I*_peak_) of the steady-state current (Figure 2). These findings reveal that under zero Ca^2+^ conditions (when a maximal driving force is applied to operate in reverse mode), NCX1.4 can overcome the Ca^2+^-depleted inactivation state (I_2_). These results differ from those previously reported for NCX1.1 [4,5,6,10,11,34]. Unlike NCX1.4, the CBD1 of NCX1.1 has 20–50 times higher Ca^2+^ affinity than that of NCX1.4 [41,42,43,44,45,46]. Therefore, even with 5 mM EGTA in the pipette solution (without added Ca^2+^), the cytosolic free Ca^2+^ concentration may reach 10–20 nM. Thus, under this “zero” Ca^2+^ condition, Ca^2+^ may fully or partially occupy the CBD1 of NCX1.1, thereby maintaining high *I*_peak_ levels. More focused research is needed to compare the reverse and forward modes of NCX1.1 and NCX1.4 under comparable “zero” Ca^2+^ conditions. Notably, the inhibitory effect of ATP or PIP_2_ on the *I*_peak_ amplitude of outward *I*_NCX_ was not observed at 0.1–10 µM cytosolic Ca^2+^ (Figure 3, Figure 4 and Figure 5), suggesting that ATP and PIP_2_ set “basal” (resting) levels of outward *I*_peak_, which can be modulated by occupation of CBD1 by Ca^2+^ (to upregulate *I*_peak_) or by occupation of CBD2 by Ca^2+^ to increase the *I*_ss_/*I*_peak_ fraction (Figure 5).

**Figure 6:**
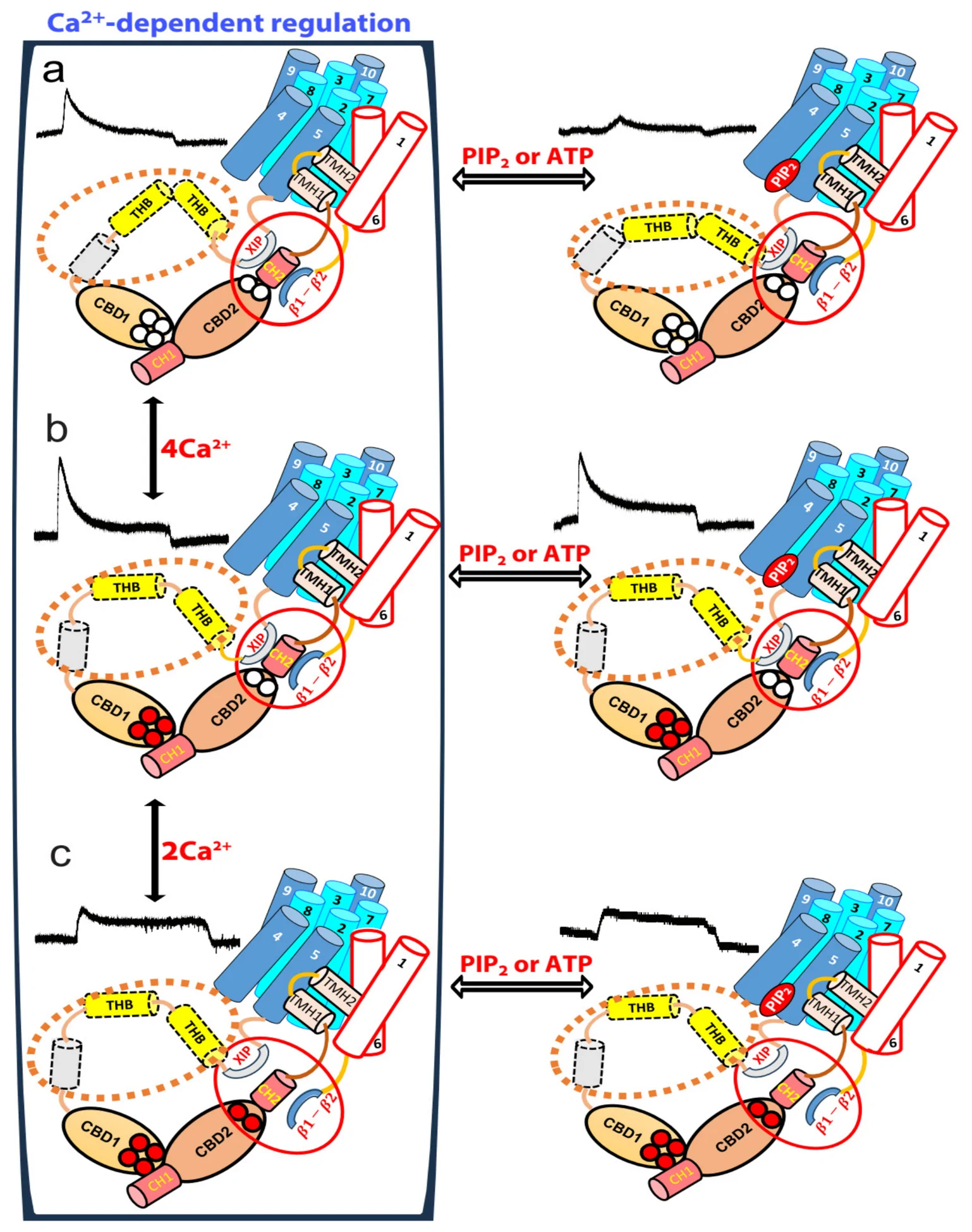
Proposed mechanism of Ca^2+^- ATP- and PIP_2_ regulatory effects on NCX1.4-mediated outward current. A schematic illustration of the proposed conformational changes associated with the sequential occupation of CBD1 and CBD2 at 0 μM (**a**), 1 μM (**b**), and 10 μM (**c**) cytosolic Ca^2+^ in the presence or absence of ATP and PIP_2_. Based on recorded NCX1.4-mediated outward currents, it is proposed that at zero cytosolic Ca^2+^ (**b**), NCX1.4 retains substantial ion-exchange activity (perhaps due to cytosolic loop–lipid membrane interactions). Adding ATP or PIP_2_ in the absence of Ca^2+^ suppresses the NCX1.4 peak current, establishing baseline activity (possibly due to destabilization of cytosolic loop–lipid membrane interactions). At 1 μM Ca^2^**^+^** (**b**), Ca^2+^ binding to CBD1 increases the NCX1.4 peak current (since the cytosolic loop–lipid membrane interactions may become more stable). Neither ATP nor PIP_2_ has a significant effect on the NCX1.4 outward current at 1 μM Ca^2+^. (**c**) At 10 μM Ca^2+^, Ca^2+^ binding to CBD2 significantly decreases NCX1.4 peak currents while increasing the fraction of steady-state current (*I*_ss_/*I*_peak_), suggesting that a probable occupancy of CBD2 by Ca^2+^ destabilizes the inactivation complex. The decrease in NCX1.4 peak currents at 10 μM Ca^2+^ can be explained by competition between Na^+^ and Ca^2+^ for a common transport site, although alternative possibilities cannot be excluded at this time. Neither ATP nor PIP_2_ affects outward NCX1.4 currents at 10 μM Ca^2+^ (when both CBDs are Ca^2+^-occupied). In panels A-C, cyan and white cylinders represent the ion-binding/transporting helices (TM2, TM3, TM7, and TM8) and the two-helix sliding bundle (TM1/TM6), respectively. Red ellipses in panels (**a**–**c**) indicate conformational changes associated with assembly and disassembly of the inhibitory complex, whereas the dashed yellow ellipses represent putative interactions between the protein cytosolic segments and the lipid membrane.

In the present study, typical *I*_ss_/*I*_peak_ values of 0.15–0.32 were observed for outward NCX currents at zero cytosolic Ca^2+^ in the absence and presence of ATP and PIP_2_ (Figure 3, Figure 4, and Figure 6a). Only Ca^2+^ binding to CBD2 increased *I*_ss_/*I*_peak_ values to 0.36–0.58 (Figure 5 and Figure 6c). These data suggest that ATP and PIP_2_ do not destabilize the inactivation β-hub/CH2 complex when both CBDs of NCX1.4 are Ca^2+^-unoccupied (Figure 6). Therefore, an alternative explanation is needed to account for the inhibitory effect of ATP and PIP_2_ on the *I*_peak_ amplitude of the NCX1.4-mediated outward current at “zero” cytosolic Ca^2+^. It was previously suggested that Ca^2+^-dependent activation of NCX1.4 (upon Ca^2+^ binding to the CBDs) may stabilize specific interactions between the cytosolic 5L6 loop and the lipid membrane [46]. Therefore, when the CBDs are Ca^2+^-unoccupied, ATP/PIP_2_ may more strongly destabilize lipid–protein interactions (Figure 6a), leading to ATP- or PIP-dependent suppression of the outward *I*_peak_ amplitude at zero free intracellular Ca^2+^ (Figure 2). In any case, the mechanism proposed for NCX1.4 under our experimental conditions differs from that of NCX1.1, in which PIP_2_-dependent destabilization of the inactivation β-hub/CH2 complex occurs (when CBD1 or both CBDs are Ca^2+^-occupied), thereby activating NCX1.1-mediated inward currents [10,34].

### 4.2. ATP and PIP_2_ May Differentially Modulate Inward and Outward Currents in NCX Variants

The observations that ATP and PIP_2_ have no significant effect on NCX1.4-mediated outward *I*_peak_ and *I*_ss_*/I*_peak_ currents at 0.1–10 µM cytosolic Ca^2+^ (Figure 3, Figure 4, and Figure 5) fundamentally differ from those for oocyte- or BHK-expressed NCX1.1 (cardiac variant) preparations, where ATP and PIP_2_ have an activating effect on inward NCX currents at 0.5–12 µM cytosolic Ca^2+^ [6,10,34,49]. This “discrepancy” warrants careful consideration when planning future experiments, because studies of ATP and PIP_2_ effects on inward and outward NCX currents used markedly different patch-clamp configurations and protein expression systems [4,5,6,10,34,49]. Therefore, additional experiments are needed to validate the differential effects of ATP and PIP_2_ on NCX1.1 outward and NCX1.4 inward currents at 0–10 µM Ca^2+^. This includes examining the effects of ATP and PIP_2_ on inward and outward NCX currents at zero cytosolic Ca^2+^ using HEK- or BHK-expressed NCX1.1 preparations, since activating effects of ATP and PIP_2_ on NCX1.1 inward currents were observed at micromolar concentrations of cytosolic Ca^2+^ in excised or oocyte-expressed NCX preparations [4,5,6,10,11,34]. In parallel, ATP and PIP_2_ effects on inward NCX1.4 currents at 0–10 µM Ca^2+^ must be analyzed in HEK and BHK expression systems.

Kinetic analyses and structure-based mutational analyses of ion fluxes, together with biophysical studies, have revealed that bidirectional ion fluxes in NCX1.1 are highly asymmetric [15,20,43,50,51]; however, no evidence has been presented for any regulatory mechanism that supports or modulates the basal functional asymmetry of these movements. The observed opposing effects of ATP and PIP_2_ on inward NCX1.1 and outward NCX1.4 currents may have primary physiological significance for the baseline “adjustment” of Ca^2+^-exit (inward current) [4,5,6,10,11,34] and Ca^2+^-entry (outward current) modes (Figure 2). For example, asymmetric regulation of NCX inward and outward currents by ATP and PIP_2_ may play a critical role in shaping neuron–glia interactions and influencing neurotransmitter release [7,8,9,52,53,54,55,56,57,58], as these mechanisms can modulate NCX’s dynamic responses to Na^+^ and Ca^2+^ transients in neurons and astroglia to match cell-specific physiological demands [3,5,6,7,8,9]. More systematic investigations are required to clarify how regulatory modes affect the forward and reverse modes across NCX variants.

### 4.3. Do ATP and PIP_2_ Represent Distinct Regulatory Modes for NCX1.4?

For nearly four decades, no physical evidence of protein kinase-mediated phosphorylation of mammalian NCX has been obtained. However, complementary studies have shown that lipid kinase-mediated phosphorylation of PI by ATP yields PIP_2_; thus, the “rapid” enzymatic formation of PIP_2_ from ATP in the presence of micromolar cytosolic Ca^2+^ can effectively activate NCX1-mediated inward currents [6,10,11,47,48,49,50]. Therefore, ATP and PIP_2_ are proposed to be part of the same pathway regulating NCX1.1, since PIP_2_ is derived from ATP via lipid kinase activity [4,5,6,34,47,48]. Our findings demonstrate that at “zero” cytosolic Ca^2+^, both ATP and PIP_2_ significantly reduce the outward peak current of HEK-expressed NCX1.4 (*I*_peak_) without altering the fraction of steady-state current (*I*_ss_/*I*_peak_) (Figure 2). These findings raise questions about whether the inhibitory effect is due to ATP-derived PIP_2_, since under the given experimental conditions (at “zero” cytosolic Ca^2+^), synthesis of PIP_2_ from ATP (via the Ca^2+^-dependent lipid kinase) is unlikely. One may ask how ATP can suppress NCX1.4 outward *I*_peak_ at zero Ca^2+^ if, under these conditions, lipid kinases do not produce PIP_2_ from ATP? Thus, ATP and PIP_2_ may represent distinct regulatory modes operating when CBDs are Ca^2+^-unoccupied.

Interestingly, distinct ATP and PIP_2_ binding sites have been identified in ATP-sensitive potassium (K_ATP_) channels, where channel opening is stimulated by PIP_2_ and inhibited by ATP through specific interactions at their respective binding sites [58,59,60,61]. Moreover, cryo-EM and functional studies show that Kir6.2 pore opening is associated with a twist of the Kir6.2 cytoplasmic domain and rotation of the N-terminal transmembrane domain of SUR1, which widens the inhibitory ATP-binding pocket and thus makes ATP binding unfavorable [59]. Because ATP and PIP_2_ may interact with different sites on NCX1.4, more focused functional and structural studies are needed to address this issue. In this respect, cryo-EM and HDX-MS methods, previously applied to NCX1.1 [10,34], NCX1.3 [11], and NCX1.4 [46], could be used to determine whether ATP binds to NCX1.4 and, if so, to resolve how the ATP-binding site interacts with other regulatory domains on NCX variants.

### 4.4. ATP/PIP_2_ Do Not Affect Ca^2+^-Dependent Effects on the Outward I_peak_ Current of NCX1.4

In the absence of ATP and PIP_2_, the outward peak current of HEK-expressed NCX1.4 increases, with no change in the *I*_ss_*/I*_peak_ ratio, as cytosolic Ca^2+^ is increased from 0 µM (where both CBDs are Ca^2+^-unoccupied) to 1 µM (where only CBD1 is Ca^2+^-occupied) (Figure 1c and Figure 2). These data show that Ca^2+^ binding to CBD1 accelerates ion-exchange currents even in the presence of ATP and PIP_2_. Notably, similar effects of Ca^2+^ binding to CBD1 were observed for inward NCX currents in oocyte-expressed NCX1.1 [10,11,33,34]. Thus, occupation of high-affinity Ca^2+^ sites on CBD1 increases peak inward and outward currents in NCX1.1 (cardiac) and NCX1.4 (brain) variants, respectively, representing a Ca^2+^-dependent activation across NCX variants [5,8,9,10,11,31,32,33,34,35].

Structure-based biophysical studies have revealed a common mechanism across NCX variants: Ca^2+^-mediated tethering of the CBDs (via Ca^2+^ binding to CBD1) rigidifies their interdomain movements, thereby enhancing TM1/TM6 sliding dynamics and promoting inward- and outward-facing conformational transitions, which, in turn, accelerate ion transport rates [5,8,10,11,12,13,62,63,64]. A complementary finding is that HEK-expressed NCX1.4 also exhibits considerably high outward peak currents at zero Ca^2+^ and at 0.1 µM cytosolic Ca^2+^ (Figure 1c, Figure 2 and Figure 3), suggesting that, under the given experimental conditions (where CBD1 is likely unoccupied), NCX1.4 retains a considerable capacity to mediate ion exchange. An interesting possibility is that ATP and PIP_2_, which decrease the peak current at zero cytosolic Ca^2+^ (Figure 1c and Figure 2), may increase Ca^2+^ affinity for CBD1, even though relatively high *I*_peak_ values are observed at 0.1 µM Ca^2+^ (Figure 1c and Figure 3).

At 1 µM Ca^2+^ (which promotes Ca^2+^ binding to CBD1), ATP and PIP_2_ have no effect on outward *I*_peak_ (Figure 3a) or on the *I*_ss_/*I*_peak_ ratio (Figure 3c), suggesting that the Ca^2+^-dependent increase in outward peak current (due to Ca^2+^ binding to CBD1) is not affected by ATP or PIP_2_. Although NCX1.1 and NCX1.4 share a common mechanism of Ca^2+^-dependent activation of the peak current upon Ca^2+^ binding to CBD1 [35,36,37,38,39,40], this event couples ATP and PIP_2_ modes differently in NCX1.1 and NCX1.4. More specifically, the difference between NCX1.1 and NCX1.4 is that ATP and PIP_2_ activate the inward NCX1.1 current when Ca^2+^ occupies CBD1 [5,6,10,34], whereas under the same conditions, ATP and PIP_2_ have no significant effect on the outward current in NCX1.4 (Figure 4).

### 4.5. ATP or PIP_2_ Do Not Inhibit Ca^2+^-Dependent Increases in the I_ss_/I_peak_ Ratio in NCX1.4

Elevated *I*_ss_/*I*_peak_ values were observed in the absence of ATP and PIP_2_ when CBD1 and CBD2 were occupied by Ca^2+^ at 10 µM Ca^2+^ (Figure 5). These observations are consistent with the notion that Ca^2+^ binding to CBD2 destabilizes the inactivation β-hub/CH2 complex (Figure 6c) [10,11,12,13]. Moreover, at 10 µM Ca^2+^ (when both CBDs are Ca^2+^-occupied), neither ATP nor PIP_2_ significantly affected the Ca^2+^-elevated *I*_ss_/*I*_peak_ values (Figure 5), suggesting that under the given experimental conditions the elevated fraction of the steady-state current is not affected by ATP or PIP_2_. Thus, NCX1.1 and NCX1.4 share a common Ca^2+^-dependent mechanism, in which the Ca^2+^-dependent increase in *I*_ss_/*I*_peak_ (upon a probable occupancy of CBD2) reflects a magnified fraction of the steady-state currents either in the presence or absence of ATP or PIP_2_. Although these results may reflect Ca^2+^-dependent alleviation of Na^+^-induced inhibition, the structure-based mechanism underlying Na^+^-induced inhibition remains unresolved for NCX1.1, NCX1.4, and all other NCX variants [3,4,5,8,49]. For example, it is unclear whether Na^+^ interacts with the transport site(s) or a regulatory site (which has not yet been identified) to cause a slow decline in peak current [5,8,36,46].

Interestingly, the NCX1.4-mediated outward peak currents recorded at 10 µM cytosolic Ca^2+^ are significantly lower than those recorded at 1 µM (Figure 1c and Figure 5). By contrast, oocyte-expressed NCX1.1 shows the opposite pattern, with ≥10 µM cytosolic Ca^2+^ elevating the *I*_peak_ of the NCX1.1-mediated inward current [10,11,30,31,32,33,34,35,36]. Several factors may contribute to the observed reduction in the *I*_peak_ of the NCX1.4 outward current at 10 µM (compared with 1 µM). Namely, the elevated cytosolic Ca^2+^ concentration may (a) decrease the driving force for Ca^2+^ influx; (b) reduce Na^+^ binding to transport sites due to Ca^2+^ competition with Na^+^; or (c) inhibit NCX-mediated ion fluxes by occupying a putative low-affinity regulatory site for Ca^2+^, the location and structural identity of which remain unresolved [49]. Conversely, in oocyte-expressed NCX1.1, increasing cytosolic Ca^2+^ from 1 µM to 10 µM increases the *I*_peak_ of the NCX1.1 forward mode (Ca^2+^ efflux), probably due to increased occupancy of the Ca^2+^ transport site and an enhanced driving force for Ca^2+^ efflux [10,11,30,31,32,33,34,35,36]. Notably, in contrast to BHK-expressed NCX1.1 [49], no “low-affinity” inhibition by Ca^2+^ has been reported in excised patches and oocyte-expressed NCX1.1 [10,11,30,31,32,33,34,35,36], meaning that some regulatory modes do not operate in these environments.

### 4.6. Alternative Mechanisms Could Be Involved in Mammalian NCX Regulation

The major findings of the present work can be summarized as follows: (a) at “zero” cytosolic Ca^2+^, NCX1.4 retains its basal outward current; (b) adding ATP or PIP_2_ at zero cytosolic Ca^2+^ inhibits the NCX1.4-mediated outward current; and (c) in a wide range of cytosolic Ca^2+^ (0.1–10 µM), there is no ATP- or PIP_2_-dependent inhibition of NCX1.4-mediated outward current. On the one hand, our findings are compatible with established mechanisms involving CBD-dependent Ca^2+^-activation of mammalian NCX [30,31,32,33,34,35,36,40], where Ca^2+^ binding to CBD1 increases the outward current *I*_peak_, whereas occupation of CBD2 increases the fraction (*I*_peak_/*I*_peak_) of steady-state ion currents (Figure 1 and Figure 6). However, previous observations for NCX1.1 cannot be reconciled with our major findings; for example, at low cytosolic Ca^2+^, the NCX1.1-mediated *I*_peak_ is negligible, whereas ATP and PIP_2_ increase *I*_peak_ [5,10,36,48,49].

Based on the compiled data, one may speculate that alternative regulatory mechanism(s) may operate alongside known regulatory modes besides CBDs and other regulatory domains. A putative regulatory protein (probably having a very high-affinity Ca^2+^ binding site on it) may interact with NCX1.4, thereby generating basal (stabilizing) conditions for NCX operation in the cellular environment. Under zero cytosolic Ca^2+^ (when the regulatory domains of NCX1.4 and the putative regulatory protein are Ca^2+^-depleted), NCX1.4 retains its basal ion-transporting activity. In this Ca^2+^-depleted state, ATP/PIP_2_ can inhibit the NCX1.4-mediated outward *I*_peak_ current, whereas Ca^2+^ binding to a putative regulatory site (outside the regulatory CBD domains) may oppose the inhibitory effect of ATP/PIP_2_. Although speculative, the proposed mechanism can explain the differential effects observed with nominally zero (Figure 2) and 0.1 µM (Figure 3) Ca^2+^. Calmodulin is a possible candidate for interaction with NCX1.4 because it is endogenously expressed in HEK293 and BHK cells, and its interaction with NCX1 [62] and NCKX4 [63] has been reported. Notably, four-point mutations at any of the conserved amino acid residues in a putative CaM-binding segment (CaMS) had differential effects on NCX1.1- and NCX1.3-mediated ion-transport activities [62]. Thus, calmodulin effects on NCX1 could be splicing-dependent. Extensive multidisciplinary research is needed to address these cardinal yet highly speculative issues.

### 4.7. Structural Variances Across NCXs May Differently Shape Regulatory Interactions

The effects of Ca^2+^ interactions with CBD1 and CBD2 on the ATP and PIP_2_ regulatory modes in distinct NCX variants warrant more focused attention in future research. One may posit that exon-dependent structural variations in Ca^2+^- and Na^+^-dependent regulation across NCX variants could underlie differential activation and deactivation of NCX forward and reverse modes by ATP and PIP_2_ regulatory modes. Notably, mutually exclusive exons (A and B) determine the number of Ca^2+^ sites at CBD2 (to control Ca^2+^-dependent alleviation of Na^+^-induced inactivation), whereas the length of the splicing segment, controlled by cassette exons (C, D, E, and F), modulates Ca^2+^ binding affinity at CBD1 to control the amplitude of the peak current [8,9,10,11,22,23,24,25,26,27,28,64,65,66]. This can explain why exon-A-containing splice variants are preferentially expressed in excitable tissues (e.g., cardiomyocytes or neurons), whereas non-excitable tissues predominantly contain exon-B variants [9,22,23,24,25,26,27,28,29,30,31,32,67,68]. Given these structure-based differences in exon-dependent ionic regulation of tissue-specific NCX1-3 splice variants [1,2,3,4,5,8,9,28,29,30,31,32], one may posit that the ATP and PIP_2_ regulatory modes are differentially activated or deactivated in tissue-specific NCXs to match the ionic and metabolic regulatory requirements of a given cell type. For example, Ca^2+^ transients may differentially activate or deactivate PIP_2_- and ATP-regulatory modes of neuronal and glial NCXs; the rationale behind this is that Na^+^-driven Ca^2+^ entry in glia (via the reverse NCX mode) can convert glia-specific Na^+^ transients into Ca^2+^ signals to couple synaptic neurotransmission [7,8,9].

Notably, cardiomyocytes only contain the NCX1.1 (ACDEF) variant, whereas neurons express multiple NCX variants, including NCX1.4 (AD), NCX1.5 (ADF), NCX2 (AC), and NCX3.2 (B) [3,5,8,40,41,42,43,54]. Thus, exon-encoded structural variations may differentially reorganize mechanism-based interactions among Ca^2+^, ATP, and PIP_2_. In particular, it remains unclear how ATP and PIP_2_ affect inward and outward *I*_NCX_ in NCX2 (which lacks Na^+^-dependent inactivation) and in NCX1.3 (which lacks Ca^2+^-dependent alleviation of Na^+^-induced inactivation due to the absence of Ca^2+^-binding sites on CBD2). A better understanding of the underlying mechanisms may enable selective pharmacological targeting of NCX variants across specific biomedical applications [3,5,8,52,53,54]. These findings provide a good platform for more focused application of cryo-EM [10,11,34], HDX-MS [16,46,66,67], and NMR [18,69,70,71,72] to resolve structural and dynamic differences that may shape regulatory specificities in tissue-specific NCX1-3 splice variants.

Some unresolved issues hinder a deeper understanding of the mechanistic interactions among NCX regulatory modes. For example, the structural details of the cytosolic TM5-CBD1 loop and the neighboring THB domain have not yet been resolved using cryo-EM [10,11,34]. This missing information is key to resolving this puzzle because Ca^2+^-dependent dissociation of the CH2/β-hub inhibitory complex, coupled with THB interaction with the lipid membrane, may activate NCX, whereas Na^+^-induced assembly of CH2/β-hub could be associated with THB detachment from the membrane, causing NCX inhibition [13,36]. Additionally, the structural details of mammalian NCX palmitoylation remain unclear, although palmitoylation-dependent conformational changes in mammalian NCX and NCKX proteins may affect interactions among distinct regulatory modes [3,5,8,73,74,75,76,77,78,79]. Further integration of structure-based regulatory mechanisms may allow selective pharmacological targeting of tissue-specific isoforms and splice variants of NCX.

## Data Availability

The original contributions presented in this study are included in the article. Further inquiries can be directed to the corresponding author.
